# Programmable Protein Stabilization with Language Model-Derived Peptide Guides

**DOI:** 10.21203/rs.3.rs-4670386/v1

**Published:** 2024-07-26

**Authors:** Lauren Hong, Tianzheng Ye, Tian Zi Wang, Divya Srijay, Lin Zhao, Rio Watson, Sophia Vincoff, Tianlai Chen, Kseniia Kholina, Shrey Goel, Matthew P. DeLisa, Pranam Chatterjee

**Affiliations:** 1. Department of Biomedical Engineering, Duke University; 2. Robert F. Smith School of Chemical and Biomolecular Engineering, Cornell University, Ithaca, NY, USA; 3. Nancy E. and Peter C. Meinig School of Biomedical Engineering, Cornell University, Ithaca, NY, USA; 4. Cornell Institute of Biotechnology, Cornell University, Ithaca, NY, USA; 5. Department of Computer Science, Duke University; 6. Department of Biostatistics and Bioinformatics, Duke University

## Abstract

Dysregulated protein degradation via the ubiquitin-proteasomal pathway can induce numerous disease phenotypes, including cancer, neurodegeneration, and diabetes. Stabilizing improperly ubiquitinated proteins via target-specific deubiquitination is thus a critical therapeutic goal. Building off the major advances in targeted protein degradation (TPD) using bifunctional small-molecule degraders, targeted protein stabilization (TPS) modalities have been described recently. However, these rely on a limited set of chemical linkers and warheads, which are difficult to generate de novo for new targets and do not exist for classically “undruggable” targets. To address the limited reach of small molecule-based degraders, we previously engineered ubiquibodies (uAbs) by fusing computationally-designed “guide” peptides to E3 ubiquitin ligase domains for modular, CRISPR-analogous TPD. Here, we expand the TPS target space by engineering “deubiquibodies” (duAbs) via fusion of computationally-designed guides to the catalytic domain of the potent OTUB1 deubiquitinase. In human cells, duAbs effectively stabilize exogenous and endogenous proteins in a DUB-dependent manner. To demonstrate duAb modularity, we swap in new target-binding peptides designed via our generative language models to stabilize diverse target proteins, including key tumor suppressor proteins such as p53 and WEE1, as well as heavily-disordered fusion oncoproteins, such as PAX3::FOXO1. In total, our duAb system represents a simple, programmable, CRISPR-analogous strategy for TPS.

## Introduction

The ubiquitin-proteasomal pathway regulates critical processes, including protein folding, DNA repair, and cell differentiation, thus helping to maintain proteostasis.^1^ Dysregulation of this pathway – such as improper degradation of tumor suppressors or mutant, misfolded proteins – can lead to severe pathogenic phenotypes, such as cancer, neurodegenerative disease, cystic fibrosis, and diabetes.^2–5^ Therefore, there is a need for proteome editing tools that are capable of correcting this dysregulation by selectively removing ubiquitin from target proteins. While the controllable installation of ubiquitin has been extensively exploited in the form of targeted protein degradation (TPD) strategies such as PROTACs, molecular glues, etc.,^1^ only recently has the reverse process, targeted protein stabilization (TPS), gained attention.^6^ Analogous to PROTACs, the current state-of-the-art TPS modality, termed deubiquitinase-targeting chimeras or DUBTACs, recruit endogenous deubiquitinases (DUBs) and rely on the design of chemical linkers, as well as the existence of small-molecule warheads, which do not exist for classically “undruggable” proteins, those that lack putative or cryptic binding site accessibility, and are conformationally disordered.^6^ Due to the labor-intensive and time-consuming process of designing *de novo* binders—whether small molecules or biologics—for new target proteins,^7^ achieving a truly programmable TPS system currently remains unrealized.

In recent years, our team has described a novel TPD strategy that involves genetically fusing target-specific short “guide” peptides, designed via sequence-based algorithms, to the ubiquitin conjugation domain of the human E3 ubiquitin ligase, CHIP.^8–12^ Without the requirement of a stable target struc2009ture, this facile, CRISPR-like design process results in chimeric proteins called “ubiquibodies” (uAbs) for TPD which can target a conformationally varied array of target proteins.^8–12^ Here, we design the analogous platform for TPS, termed deubiquibodies (duAbs), by fusing computationally-designed peptide guides to the catalytic domain of the potent OTUB1 deubiquitinase. Utilizing pre-existing binders, our first-generation fusion duAb architecture effectively stabilized exogenous and endogenous proteins in a DUB-dependent manner following ectopic expression in human cells. We then showcase the inherent programmability of duAbs by swapping in new target-binding peptides designed via our recent generative language models, SaLT&PepPr, PepPrCLIP, and PepMLM.^10–12^ These novel duAbs stabilize their intended target substrates, including the transcription factors β-catenin and FOXP3, the tumor suppressors WEE1 and p53, and a disordered fusion oncoprotein PAX3::FOXO1, thereby demonstrating the ease and speed with which new duAbs can be designed to diverse proteins in the proteome.

## Results

### Engineering a potent DUB-dependent stabilization system

**Figure 1.**
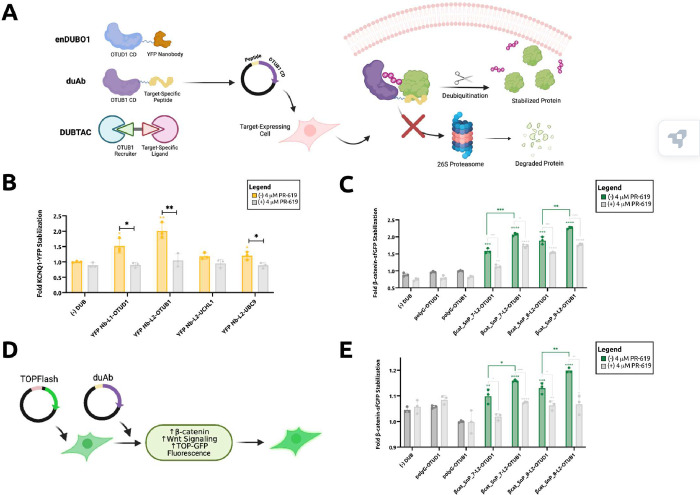
Engineering of the duAb architecture. (A) Targeted protein stabilization strategies. enDUBO1 consists of a YFP nanobody (Nb) fused to the OTUD1 deubiquitinase catalytic domain (CD).^13^ The duAb architecture consists of a target-specific peptide fused to the OTUB1 deubiquitinase CD. DUBTACs are heterobifunctional molecules made up of a target-specific small molecule ligand linked to a covalent ligand EN523, which recruits the endogenous OTUB1 deubiquitinase. EN523 induces proximity between OTUB1 and a ubiquitinated target.^6^ (B) KCNQ1-YFP stabilization by YFP Nb-based stabilizers in HEK293T cells determined by flow cytometric analysis. Cells were co-transfected with a pCMV-NEDD4L vector in the presence or absence of 4 μM PR-619 DUB inhibitor as indicated. Data are the average of independent replicates (*n* = 3). L1 = GAPGSG, L2 = GSGSG. (C) β-catenin-sfGFP stabilization in HEK293T cells measured by flow cytometric analysis. Cells were transiently transfected in the presence or absence of 4 μM PR-619 DUB inhibitor. Data are the average of independent replicates (*n* = 3). (D) TOP-GFP assay for quantifying Wnt signaling in HEK293T cells.^19^ Stabilization of endogenous β-catenin results in higher levels of Wnt signaling and increased GFP levels, measured by flow cytometry. (E) TOP-GFP signals in HEK293T cells measured by flow cytometric analysis. Cells were transiently transfected in the presence or absence of 4 μM PR-619 DUB inhibitor. Data are the average of independent replicates (*n* = 3). For (B)-(E), normalized cell fluorescence was calculated by dividing the percentage of sfGFP+ cells in a sample by that of control cells: (−) DUB with no DUB inhibitor for (B), and polyG-OTUB1 with no DUB inhibitor for (C) and (D). Statistical analysis was performed using the two-tailed Student’s *t* test using GraphPad Prism 10 software, with calculated *p* values are represented as follows: *, *p* ≤ 0.05, **, *p* ≤ 0.01, ***, *p* ≤ 0.001, ****, *p* ≤ 0.0001. Samples with *p* value representations above their respective bars reflect comparisons between the control and that sample; all other *p* value notations compare those specific samples.

Recently, Kanner et al. fused the OTUD1 deubiquitinase domain to yellow fluorescent protein-targeting nanobodies (YFP Nbs) to create enDUBO1 constructs that stabilize target-YFP fusion proteins (Figure 1A).^13^ We hypothesized that DUB domains exhibiting more potent deubiquitinase activity may improve TPS. To evaluate potential effectors for recruitment, Poirson et al., conducted a proteome-scale induced proximity screen to rank both ubiquitinating and deubiquitinating enzymes in terms of catalytic activity.^14^ They isolated a subset of deubiquitinases, including OTUB1 and UCHL1, as well as a SUMOlase, UBC9, with potent stabilization activity (Supplementary Table 1).^14^ Of note, OTUB1 is the endogenous deubiquitinase recruited by DUBTACs (Figure 1A).^6^

Using known domain annotations of these proteins in UniProt,^15^ we isolated the catalytic domains of each enzyme and fused them to the aforementioned YFP Nbs via either the GAPGSG linker (used for enDUBO1) termed L1^13^ or the flexible GSGSG linker already used in the uAb architecture termed L2 (Supplementary Table 1 and 2). To evaluate these designs, we employed a reporter fusion between the potassium ion channel protein, KCNQ1, and YFP, which was co-transfected in HEK293T cells with KCNQ1’s E3 ubiquitin ligase, Nedd4L.^13,16^ Our results showed that the YFP Nb-L2-OTUB1 fusion significantly increased KCNQ1-YFP levels, which exceeded the stabilization measured for enDUBO1 (YFP Nb-L1-OTUD1), YFP Nb-L2-UCHL1, and YFP Nb-L2-UBC9. We additionally sought to determine whether our DUB fusions acted in a DUB-dependent manner by employing the pan-DUB inhibitor PR-619.^17^ Importantly, addition of PR-619 at a standard concentration (4 μM) was observed to abrogate stabilization, confirming the DUB-dependent mechanism of these stabilizer constructs (Figure 1B).

To enable the generation of a peptide-guided stabilization system, analogous to our uAb constructs for degradation, we sought to leverage our existing computationally-designed peptides as target-recruiting “guides”. As a first candidate, we chose the β-cat_SnP_7 and β-cat_SnP_8 peptides derived from our SaLT&PepPr algorithm, both of which exhibit nanomolar binding affinity to β-catenin.^10^ Our hypothesis was that fusing these peptides to a potent deubiquitinase catalytic domain would induce stabilization of β-catenin in HEK293T cells, which possess an intact Wnt signaling pathway including expression of β-catenin’s natural E3 ubiquitin ligase, β-TrCP.^18^ We demonstrate that, when fused to β-cat_SnP_7 and β-cat_SnP_8 via L2, the OTUB1 catalytic domain exhibits statistically significant stabilization of β-catenin-sfGFP proteins compared to the OTUD1 catalytic domain (Figure 1C). We again show that employing the pan-DUB inhibitor PR-619 at a standard 4 μM concentration inhibits DUB-dependent stabilization of β-catenin-sfGFP, as expected. We further corroborated this result by co-transfecting β-cat_SnP_7-L2-OTUB1 and β-cat_SnP_8-L2-OTUB1 fusions into HEK293T cells alongside TOP-GFP, a fluorescent reporter that serves as a reliable readout of β-catenin–dependent transcriptional activity (Figure 1D).^19^ Cells transfected with β-cat_SnP_7-L2-OTUB1 and β-cat_SnP_8-L2-OTUB1 exhibited significantly higher Wnt signaling than either untransfected cells or cells transfected with β-cat_SnP_7-L2-OTUD1 and β-cat_SnP_8-L2-OTUD1 (Figure 1E), consistent with the β-catenin-sfGFP stabilization observed above and indicative of increased β-catenin levels. Given these results, we refer to binder-L2-OTUB1 fusions as deubiquibodies, or duAbs (Figure 1A).

### Demonstrating duAb programmability

**Figure 2.**
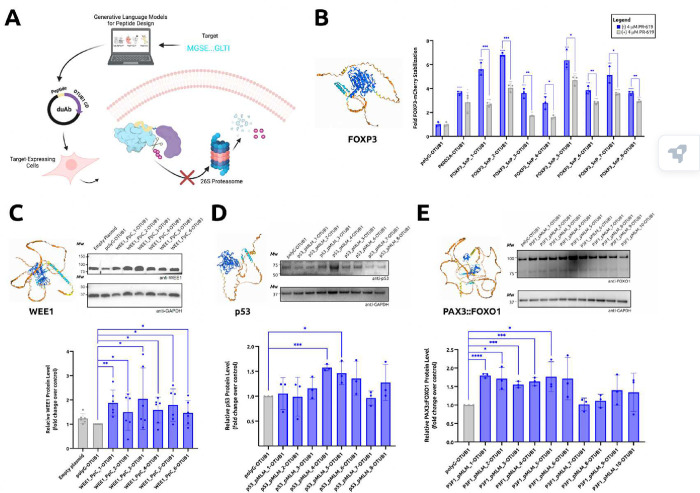
Programmable target stabilization via language model-derived peptides. (A) Programmable target stabilization via language model-derived peptides. (B) FOXP3-mCherry stabilization in HEK293T cells. Cellular mCherry fluorescence was measured by flow cytometry in independent replicates (*n* = 3). Normalized cell fluorescence was calculated by dividing the percentage of mCherry+ cells in a sample by that of control cells expressing a duAb vector expressing a non-specific poly-glycine (polyG) control peptide sequence. (C) Stabilization of endogenous WEE1 in protein extracts of HepG2 cells analyzed by immunoblotting. Cells were transiently transfected with a pCMV plasmid encoding a polyG-OTUB1 control or one of the peptide-guided OTUB1 constructs as indicated. Transient transfection with an empty pCMV plasmid served as an additional control. Blots were probed with anti-WEE1 and anti-GAPDH antibodies and are representative of biological replicates (*n* = 3) and technical replicates (*n* = 2) with similar results. (D) Stabilization of endogenous p53 in protein extracts of HeLa cells analyzed by immunoblotting. HeLa cells were transiently transfected with a pCMV plasmid encoding one of the candidate duAbs while transfection with a polyG peptide-guided duAb served as a control. An equivalent amount of protein was loaded in each lane. Blots were probed with anti-p53 and anti-GAPDH antibodies and are representative of biological replicates (*n* = 3). (E) Stabilization of endogenous PAX3::FOXO1 in protein extracts of RH4 cells analyzed by immunoblotting. RH4 cells were transiently transfected with a pCMV plasmid encoding one of the candidate duAbs while transfection with a polyG peptide-guided duAb served as a control. Blots were probed with anti-FOXO1 and anti-GAPDH antibodies and are representative of biological replicates (*n* = 3). For all immunoblots in (C)-(E), an equivalent amount of protein was loaded in each lane. Molecular weight (*M*_W_) ladder is indicated at left. Intensity of target protein bands was calculated via densitometry and normalized to intensity of GAPDH loading control and then normalized to polyG-OTUB1 control. Data are the average of biological replicates and technical replicates (*n* = 6 for WEE1 and *n =* 3 for p53 and PAX3::FOXO1). Statistical analysis for this figure was performed using the two-tailed Student’s *t* test using GraphPad Prism 10 software, with calculated *p* values are represented as follows: *, *p* ≤ 0.05, **, *p* ≤ 0.01, ***, *p* ≤ 0.001, ****, *p* ≤ 0.0001. The *p* values above each bar in the fold stabilization and densitometry analyses represent the comparison between the control (polyG-OTUB1, no DUB inhibitor) and the respective sample; all other *p* value notations compare the specified samples. All structures were predicted via the AlphaFold3 server, and the shading was done according to AlphaFold’s confidence metric, plDDT, as follows: Very low (plDDT < 50) = Orange, Low (70 > plDDT > 50) = Yellow, Confident (70 > plDDT > 90) = Light Blue, Very high (plDDT > 90) = Light Blue.

The enDUBO1 fusion, described previously, relies on YFP tagging of target proteins to enable YFP Nb binding and subsequent OTUD1-mediated deubiquitination,^13^ limiting the applicability of this genetically-encoded platform. Having shown that our duAb architecture is compatible with guide peptide binder sequences for target-specific stabilization, we sought to demonstrate duAb programmability by designing new guide peptides to conformationally-diverse target proteins. Specifically, this involved generative peptide design language models, which are not constrained by the requirement of stable tertiary structures (Figure 2A). We first focused our attention on FOXP3, a classically undruggable transcription factor that plays a central role in the development and function of regulatory T cells (Tregs).^20^ FOXP3 is naturally regulated by the CHIP E3 ubiquitin ligase, which is expressed in HEK293T cells.^21^ We applied our SaLT&PepPr interface-prediction algorithm to isolate guide peptides from its well-known interacting partner, NFAT (Supplementary Table 2)^21,22^ and subsequently tested the peptide-guided duAbs in a FOXP3-mCherry HEK293T stable cell line. Our results demonstrated that SaLT&PepPr-derived duAbs induced statistically significant stabilization of FOXP3-mCherry in a DUB-dependent manner, and outperformed a duAb composed of a previously-designed P60D2A FOXP3-targeting peptide (Figure 2B).^23^

Encouraged by the stabilization of FOXP3, we moved on to WEE1, an inhibitor of tumor growth in non-cancerous eukaryotic somatic cells that acts as a kinase to phosphorylate the cyclin-dependent kinase (CDK1)–cyclin B1 complex.^24^ This phosphorylation hinders the advancement of cell cycle in the S and G2 phases of mitosis.^24^ It has been established that WEE1 is regulated by the ubiquitin-proteasomal pathway in hepatocellular carcinoma cell lines and that treatment with a proteasome inhibitor or DUBTAC leads to WEE1 stabilization in these cells.^6,25,26^ To target WEE1 for stabilization using our duAb technology, we designed 8 guide peptide specific for WEE1: 2 via SaLT&PepPr and 6 via our *de novo* peptide design algorithm, PepPrCLIP (Supplementary Table 2).^10,11^ The resulting guide peptides were each fused to OTUB1 in our duAb plasmid and tested in HepG2 hepatocellular carcinoma cells. Immunoblot analysis with an anti-WEE1 antibody revealed that each of our peptide-guided duAbs induced statistically-significant stabilization of endogenous WEE1 (Figure 2C), motivating future comparison to DUBTACs for druggable and undruggable targets.^6^

We next sought to stabilize p53, a key tumor suppressor protein that regulates cell cycle arrest, apoptosis, and DNA repair in response to cellular stress and DNA damage.^27^ The ability to stabilize p53 with duAbs would ensure its availability to suppress tumor formation and growth by maintaining genomic integrity and inhibiting malignant cell proliferation.^28^ p53 is largely disordered (Figure 2D), thus we designed eight peptides using our most recent peptide generator, PepMLM, which only requires the target sequence as input.^12^ PepMLM demonstrates a nearly 40% hit rate for binder design, which is higher than structure-based binder design methods like RFDiffusion.^12^ As p53 is destabilized via ubiquitination in pediatric alveolar rhabdomyosarcoma (ARMS), amongst many other cancers, we transfected an ARMS cell line (RH4) with plasmid DNA encoding 8 different duAb designs.^29^ Immunoblot analysis revealed that two of our duAbs, p53_pMLM_4 and p53_pMLM_6, exhibited potent duAb-dependent stabilization as evidenced by significant increases in p53 levels (Figure 2D).

Finally, it has been reported that fusion oncoproteins which drive pediatric cancers, such as EWS::FLI1 for Ewing sarcoma, exhibit a “Goldilocks” phenomenon, where suppression of their ubiquitination can induce fusion oncoprotein overdose and cancer cell death.^30^ However, pharmacologically stabilizing these proteins is highly difficult, as these proteins exhibit almost complete structural disorder with no discernable binding pockets (Figure 2E).^31^ To overcome this structural disorder, we again used our target sequence-conditioned PepMLM algorithm to generate 10 guide peptides to PAX3::FOXO1, the main driver of pediatric ARMS.^32^ After transfecting plasmids encoding these peptide-guided duAbs into fusion-positive RH4 ARMS cells, we observed stable increases in the levels of both PAX3::FOXO1 fusion oncoprotein and FOXO1 for 5 of the duAb designs (Figure 2E). Overall, our results demonstrate the facile programmability of duAbs to stabilize proteins with diverse tertiary structures and functional properties.

## Discussion

The CRISPR-Cas system is incredibly powerful for genome editing due to its modularity. The simple design of an sgRNA to a new genomic sequence enables Cas-mediated editing of that locus.^33^ However, CRISPR is not limited to just gene disruption or inhibition – the system has been extended to conduct single base editing, gene insertion, and even gene activation via fusion to transactivation domains.^33^ Analogous to the RNA-guided CRISPR inhibition (CRISPRi) system, our peptide-guided uAb degraders are genetically-encodable “off” switches for proteins. Here, we have created the respective “on” switch (analogous to CRISPRa), by developing peptide-guided duAbs for TPS. Integrated with our rapid binder generation algorithms, our results demonstrate the simplicity and programmability of duAbs to stabilize diverse target substrates intracellularly.

As duAbs are ~290 amino acids in length (Supplementary Table 1), their intracellular delivery, at first glance, poses a challenge for therapeutic application. However, with the rapid advancements of targeted lipid nanoparticle (LNP) platforms,^34^ duAbs can be readily encapsulated as mRNA and delivered to specific tissues of interest, as opposed to DUBTACs, which may home to any tissue, risking potential side effects and toxicity concerns. More interestingly, as a genetically-encoded tool, peptide-guided duAbs, alongside uAbs, may together comprise a powerful proteome screening tool for drug discovery, allowing for combinatorial protein activation and inhibition screening, similar to the CRISPR-based Perturb-Seq platform.^35^ Finally, with recent advancements in protein representation algorithms, we envision that our language model-generated peptides can be augmented to specifically bind post-translational and mutant isoforms of target proteins,^36,37^ and can be fused to other PTM domains, including kinases, phosphatases, and deglycosylases, to name a few.^7^ This study, enabling modular peptide-guided protein stabilization, represents a next step towards this eventual goal of a fully programmable proteome editing system.

## Materials and Methods

### Binder design

The β-cat_SnP_7 peptide,^10^ P60D2A peptide,^23^ and YFP nanobodies^13^ were described in previous works and obtained from respective manuscript metadata. Novel binding peptides designed in this study were either generated by the previously-described SaLT&PepPr algorithm^10^ (https://huggingface.co/ubiquitx/saltnpeppr) via input of an interacting partner sequence, by the *de novo* PepPrCLIP algorithm^11^ (https://huggingface.co/ubiquitx/pepprclip) via input of the target protein sequence, or by the target sequence-conditioned PepMLM algorithm (https://huggingface.co/ChatterjeeLab/PepMLM-650M). All binder sequences can be found in Supplementary Table 2.

### Generation of plasmids

All duAb plasmids were generated from the standard pcDNA3 vector, harboring a cytomegalovirus (CMV) promoter and a C-terminal P2A-eGFP cassette. An Esp3I restriction site was introduced immediately upstream of the OTUB1 catalytic domain and flexible GSGSG linker via the KLD Enzyme Mix (NEB) following PCR amplification with mutagenic primers (Azenta). For duAb assembly, oligos for candidate peptides were annealed and ligated via T4 DNA Ligase into the Esp3I-digested duAb backbone. Assembled constructs were transformed into 50 μL NEB Turbo Competent *Escherichia coli* cells, and plated onto LB agar supplemented with the appropriate antibiotic for subsequent sequence verification of colonies and plasmid purification (Azenta).

### Cell culture

The HEK293T cell line was maintained in Dulbecco’s Modified Eagle’s Medium (DMEM) supplemented with 100 units/mL penicillin, 100 mg/mL streptomycin, and 10% fetal bovine serum (FBS). The hepatocellular carcinoma cell line, HepG2, was maintained in Eagle’s Minimum Essential Medium (EMEM) supplemented with 100 units/mL penicillin, 100 mg/mL streptomycin, and 10% fetal bovine serum (FBS). The alveolar rhabdomyosarcoma cell line, RH4, was maintained in RPMI 1640 supplemented with 100 units/mL penicillin, 100 mg/mL streptomycin, and 10% fetal bovine serum (FBS). The RH4 cell line was a generous gift from Dr. Corinne Linardic. For duAb screening in reporter cell lines, pcDNA-duAb (500 ng) plasmids were transfected into cells as triplicates (2×10^5^/well in a 24-well plate) with Lipofectamine 2000 (Invitrogen) in Opti-MEM (Gibco). After 2 days post transfection, 4 μM PR-619 (DUB inhibitor) was added to applicable cells (with equivalent volume of media added to non-treated cells), and subsequently cells were harvested within 24 hours post-treatment and analyzed on a Attune NxT Flow Cytometer (ThermoFisher) for GFP fluorescence (488-nm laser excitation, 530/30 filter for detection) and mCherry fluorescence (561-nm laser excitation, 620/15 filter for detection). 10,000 events were gated for data analysis based on default FSC/SSC parameters for the analyzed cells. Cells expressing eGFP and mCherry were gated, and these were normalized to a sample transfected with a non-targeting duAb using the FlowJo software (https://flowjo.com/). Representative flow cytometry gating strategies are indicated in Supplementary Figure 1. For endogenous target screening in cell lines, pcDNA-duAb (800 ng) plasmids were transfected into cells as duplicates (3×10^5^/well in a 12-well plate) with Lipofectamine 2000 (Invitrogen) in Opti-MEM (Gibco). Cells were harvested after 72 hours for subsequent cell fractionation and immunoblotting.

### Lentiviral production

For target-reporter packaging, HEK293T cells were seeded in a 6-well plate and transfected at ~50% confluency. For each well, 0.5 μg pMD2.G (Addgene #12259), 1.5 μg psPAX2 (Addgene #12260) and 0.5 μg of the target-mCherry reporter transfer vector were transfected with Lipofectamine 3000 (Invitrogen) according to the manufacturer’s protocol. The medium was exchanged 8 hours post transfection, and the viral supernatant was harvested at 48 and 72 hours post-transfection. The viral supernatant was concentrated to 100x in 1x DPBS using Lenti-X Concentrator (Clontech, 631232) according to the manufacturer’s instruction, and stored at −80°C for further use.

### Target-mCherry reporter monoclonal cell line generation

For target-reporter cell line generation, 1×10^5^ HEK293T cells were mixed with 20 μL of the concentrated virus in a 6-well plate. Media was changed 24 hours post transduction. Antibiotic selection was started 36 hours post transduction by adding 2 μg/mL puromycin (Sigma, P8833). Cells were harvested for sorting at 5 days post antibiotic selection, and a single cell of mCherry positive was plated in a 96 well plate. Genomic PCR was performed after cell growth to validate the genotype of the monoclonal cell line.

### Functional Assays

For the TOP-GFP assay, 2×10^5^ HEK293T cells/well were seeded on a 24-well plate 20–24 hours prior to transfection. On the day of transfection, each well received the following plasmids: TOP-GFP plasmid (Addgene plasmid # 35489) and a duAb plasmid. A total of 500 ng of plasmid DNA in a ratio of TOP-GFP:duAb plasmids = 1:1 was mixed with Lipofectamine 2000 reagent in serum-free Opti-MEM medium and added dropwise to each well after incubation at room temperature for 20 min. After 72 h of incubation, cells were harvested and analyzed similarly as mentioned for duAb screening. Viable, single cells were gated, and normalized EGFP cell fluorescence was calculated as compared to a sample transfected with a non-targeting duAb, using the FlowJo software (https://flowjo.com/).

### Cell fractionation and immunoblotting

On the day of harvest, cells were detached by addition of 0.05% trypsin-EDTA and cell pellets were washed twice with ice-cold 1X PBS. Cells were then lysed and subcellular fractions were isolated from lysates using a 1:100 dilution of protease inhibitor cocktail (Millipore Sigma) in Pierce RIPA buffer (ThermoFisher). Specifically, the protease inhibitor cocktail-RIPA buffer solution was added to the cell pellet, the mixture was placed at 4 °C for 30 min followed by centrifugation at 15,000 rpm for 10 min at 4 °C. The supernatant was collected immediately to pre-chilled PCR tubes and quantified using the Pierce BCA Protein Assay Kit (ThermoFisher). 20 μg lysed protein was mixed with 4X Bolt^™^ LDS Sample Buffer (ThermoFisher) with 5% β-mercaptoethanol in a 3:1 ratio and subsequently incubated incubated at 95 °C for 10 min prior to immunoblotting, which was performed according to standard protocols. Briefly, samples were loaded at equal volumes into Bolt^™^ Bis-Tris Plus Mini Protein Gels (ThermoFisher) and separated by electrophoresis. iBlot^™^ 2 Transfer Stacks (Invitrogen) were used for membrane blot transfer, and following a 1 h room-temperature incubation in 1% BSA blocking buffer, proteins were probed with rabbit anti-WEE1 antibody (Abcam, Cat # ab137377, diluted 1:1000), mouse anti-p53 antibody (Santa Cruz Biotechnology, Cat # sc-126, diluted 1:1000), rabbit anti-FOXO1 antibody (Cell Signaling Technology, Cat # 2880S, diluted 1:1000), or mouse anti-GAPDH (Santa Cruz Biotechnology, Cat # sc-47724; diluted 1:10000) for overnight incubation at 4 °C. The blots were washed three times with 1X TBST for 5 min each and then probed with a secondary antibody, donkey anti-rabbit IgG (H+L), horseradish peroxidase (HRP) (Abcam, Cat # ab7083, diluted 1:5000) or goat anti-mouse IgG (H+L) Poly-HRP (ThermoFisher, Cat # 32230, diluted 1:5000) for 1–2 h at room temperature. Following three washes with 1X TBST for 5 min each, blots were detected by chemiluminescence using a BioRad ChemiDoc^™^ Touch Imaging System (Biorad). Densitometry analysis of protein bands in immunoblots was performed using ImageJ software as described here: https://imagej.nih.gov/ij/docs/examples/dot-blot/. Briefly, bands in each lane were grouped as a row or a horizontal “lane” and quantified using FIJI’s gel analysis function. Intensity data for the duAb bands was first normalized to band intensity of GAPDH in each lane then to the average band intensity for empty duAb vector control cases across replicates.

### Structure prediction

All structures were predicted via the AlphaFold3 server (https://alphafoldserver.com/), and the shading was done according to AlphaFold’s confidence metric, plDDT, as follows: Very low (plDDT < 50) = Orange, Low (70 > plDDT > 50) = Yellow, Confident (70 > plDDT > 90) = Light Blue, Very high (plDDT > 90) = Light Blue.

### Statistical analysis and reproducibility

Sample sizes were not predetermined based on statistical methods but were chosen according to the standards of the field (at least three independent biological replicates for each condition), which gave sufficient statistics for the effect sizes of interest. All data were reported as average values with error bars representing standard deviation (SD). Statistical analysis was performed using the two-tailed Student’s *t* test using GraphPad Prism 10 software, with calculated *p* values are represented as follows: *, *p* ≤ 0.05, **, *p* ≤ 0.01, ***, *p* ≤ 0.001, ****, *p* ≤ 0.0001. The *p* value representations above each bar in the fold stabilization and densitometry analyses are indicative of comparisons between the control and the respective sample; all other *p* value notations are between the specified samples. No data were excluded from the analyses. The experiments were not randomized. The investigators were not blinded to allocation during experiments and outcome assessment.

## Data Availability

All data needed to evaluate the conclusions in the paper are present in the paper and supplementary tables. Raw data underlying graphical figures are provided as Source Data. The duAb cloning vector will be deposited to Addgene upon publication. All raw and processed data (including raw immunoblots) have been deposited to the Zenodo repository: https://doi.org/10.5281/zenodo.12602334.
